# Reliability of sonographic fetal weight estimation in triplet pregnancies: a retrospective cohort study

**DOI:** 10.1007/s00404-018-4746-0

**Published:** 2018-03-17

**Authors:** Sophie Pils, Stephanie Springer, Rudolf Seemann, Verena Wehrmann, Christof Worda, Johannes Ott

**Affiliations:** 1 Clinical Division of Gynecologic Endocrinology and Reproductive Medicine, Department of Obstetrics and Gynecology, Medical University of Vienna, Waehringer Guertel 18-20, 1090 Vienna, Austria; 2 Clinical Division of Obstetrics and Fetomaternal Medicine, Department of Obstetrics and Gynecology, Medical University of Vienna, Waehringer Guertel 18-20, 1090 Vienna, Austria; 3 Department of Craniomaxillofacial and Oral Surgery, Medical University of Vienna, Waehringer Guertel 18-20, 1090 Vienna, Austria

**Keywords:** Fetal weight, Birth weight, Multiple pregnancy, Triplet pregnancy, Ultrasound

## Abstract

**Purpose:**

To review our experience in ultrasound fetal weight estimation in our large population of triplet pregnancies.

**Methods:**

Ninety-seven triplet pregnancies were retrospectively included between January 2003 and January 2017. Sonographic fetal weight estimation using Hadlock’s and Schild’s formulas was compared to actual birth weight in a tertiary-care center in Vienna, Austria. Statistical analyses were performed using a stepwise linear regression model and crosstabs.

**Results:**

The median discrepancy between the sonographically estimated fetal weight by Hadlock’s formula and the actual birth weight was 106 g (IQR 56–190). The percentage error and its standard deviation were − 2.5 ± 12.1%, and the median percentage error was − 3.6%. Concerning the use of Hadlock’s formula, estimated fetal weight was the most important factor predictive of actual birth weight with an estimate of 0.920 (*p *< 0.001). Female neonates had been overestimated by a mean of 50.473 g per fetus. The sonographic prediction of small-for-gestational-age neonates was significantly reliable (*p* < 0.001), with positive and negative predictive values ranging from 81.3 to 100.0%. Similar results were obtained for Schild’s formula.

**Conclusion:**

Even if sonographically estimated fetal weight in triplet pregnancies has a high overall accuracy of fetal weight estimation, there are some limitations in prediction of intrauterine growth restrictions, especially in female fetuses.

## Introduction

In times of increasing number of multiple pregnancies that also include triplets [1], clinical knowledge of these issues needs to be expanded. Exact sonographic estimation of fetal weight before delivery is essential in these pregnancies for several reasons: triplet pregnancies are associated with high rates of preterm deliveries [2–5]; and the teams on the neonatal intensive care units need the information before delivery to be accurately prepared for postnatal treatment. Moreover, there is an increased incidence of fetal growth restriction in triplets [6], which is linked to higher risks for preterm delivery, poor perinatal outcomes, and increased mortality and morbidity [7, 8]. And, last but not least, birth-weight discordance is significantly associated with fetal and neonatal mortality in both twin and triplet pregnancies [9].

Ultrasound is the only tool to predict fetal weight. Accuracy depends on clinical experience, surrounding factors including the time interval between weight estimation and delivery, and quality of the equipment [10]. Moreover, the performance of ultrasound could be seen as more difficult in triplets. Empirically, especially non-experts can experience troubles in fetal sonography due to higher risk of malpresentation and double measurements. In addition, birth-weight discordance is common among multiple pregnancies [6]. Thus, the question arises whether ultrasound is a reliable method in triplet pregnancies. Evidence about the accuracy of sonographically estimated fetal weight in triplet pregnancies is scarce [8–13]. Although good correlations between estimated fetal weight and actual birth weight have been reported [12], the reliability for the identification of growth-restricted fetuses seems suboptimal [8, 13].

Because only a few studies have been published that have addressed this issue directly, providing more evidence seems warranted. Thus, we aimed to present an analysis of our clinical experience with sonographic fetal weight estimation and its accuracy in our large, retrospective population of triplet pregnancies. The focus was also on factors that add to biometry’s predictive value for an accurate estimation of the actual birth weight, as well as on the reliability to predict small-for-gestational-age (SGA) neonates and growth discrepancy.

## Materials and methods

### Patient population and study design

In this retrospective analysis, we studied 135 women who were diagnosed with a triplet pregnancy at the time of the first trimester screening from January 2003 to January 2017. The following patients were then excluded from the study: seven women did not deliver at the department and had no follow-up and there were missing data in ten women. Moreover, we excluded all women who underwent multifetal pregnancy reduction (*n* = 15) or had an intrauterine fetal death (IUFD) of at least one fetus before the onset of viability (*n* = 6). This means that, only cases that started with triplets and gave birth to triplets were included. This resulted in a final study population of 97 triplet pregnancies with 291 fetuses/neonates for this analysis. Parts of these data have been published previously with a focus on serial cervical length measurements [14].

As reported previously [14–16], a screening program for pregnant women at perceived risk of preterm delivery, which includes multiple pregnancies, has been established for many years at the Department of Maternal–Fetal Medicine of the Medical University of Vienna, Austria. The department is the reference center for maternal–fetal medicine in Eastern Austria and the annual number of deliveries was at least 2500 during the study period. The screening program included fetal biometry using Hadlock’s formula [17] and cervical length measurement by transvaginal ultrasound every 2 weeks from week 16 + 0 until delivery. All ultrasound examinations were performed by highly experienced obstetricians, all members of the clinical working group for multiple pregnancies, and were performed on the same two ultrasound devices. A Toshiba Power Vision (Toshiba, Tokyo, Japan) ultrasound machine was used until 2010, and a Toshiba Aplio MX (Toshiba, Tokyo, Japan) machine since 2010.

The basic perinatology database at the department uses the Viewpoint^®^ software (GE Healthcare, Wessling, Germany), which was also used for data acquisition. In a retrospective data set, it can be considerably difficult to correlate each fetus examined prenatally with the actual birth order. We used the same matching criteria as Weissman et al.: “matching was performed according to the last ultrasound examination performed before delivery with the details of birth order, position in the uterus prior to delivery, and presentations reported. Dissimilar sex in any given set was also used for identification. If in a given set discordancy existed in one fetus, the discordant newborn was matched to the discordant measurement performed prenatally” [12].

This study was approved by the Institutional Review Board of the Medical University of Vienna (IRB number: 1602/2015) on 14th April 2016, and was valid for 1 year after approval. The study protocol was in accordance with the Helsinki Declaration and current Austrian law and, thus, neither written nor verbal informed consent was necessary according to the Ethics Committee of the Medical University of Vienna. Therefore, it has not been obtained. The data were de-identified for statistical analysis.

### Parameters analyzed

The main outcome parameters were: the last fetal weight estimated before delivery, the actual birth weight, and the discrepancy between these two parameters. For categorization of these parameters according to percentiles, reliable reference populations are needed. At our department, sonographic fetal weight estimation is based on Hadlock’s formula [17]. Additionally, we calculated fetal weight estimation using Schild’s sex-specific formula to evaluate whether a sex-specific formula would increase the accuracy of fetal weight estimation [18]. For each fetus, fetal growth restriction (FGR) was defined as estimated fetal weight < 10th percentile for gestational age, using for reference the (i) Canadian Perinatal Surveillance System singleton growth curves [19], which was published in 2001, which also included a western population and was also used in the most recent article on fetal weight estimation in triplets [13]. (ii) The Percentile Values for the Anthropometric Dimensions of Triplet Neonates in Germany [20] was also used, published in 2016. Similarly, for each fetus, SGA was defined as actual birth weight < 10th percentile using the same reference population.

According to a recent analysis on fetal weight estimation in triplets [13], we also focused on inter-triplet growth discordance > 25%. Thus, for each pregnancy, estimated fetal weight discordance (%) was defined as (largest triplet estimated fetal weight − smallest triplet estimated fetal weight)/(largest triplet estimated fetal weight) × 100; and actual birth-weight discordance (%) was defined as (largest triplet actual birth weight − smallest triplet actual birth weight)/(largest triplet actual birth weight) × 100.

In addition, the following parameters were included: gestational age at delivery, categorized into ≤ 27 and ≥ 28 completed weeks for the multivariate analysis; the time interval between the last biometry and delivery; maternal age at delivery; parity; pregnancies after in vitro fertilization (IVF); cigarette smoking; and chorionicity categorized into mono-/dichorionic and trichorionic for the multivariate analysis. All patients were delivered by Caesarean section. All newborns were examined by a senior neonatologist, who also confirmed that there were no malformations present.

### Statistical analyses

Nominal variables are reported as numbers and frequencies, and continuous variables as medians and interquartile ranges (IQR). To compare nominal variables between groups, the Fisher’s exact test was applied. The accuracy of weight estimation is described by the percentage error and its standard deviation and the median percentage error.

For crosstabs; odds ratio, sensitivity, specificity, and positive and negative predictive values are provided with their 95% confidence intervals (CI). These analyses were performed using SPSS statistics for Windows, version 24.0 (SPSS Inc., Chicago, USA). To evaluate factors that were predictive of the actual birth weight, a generalized linear model with a Poisson distribution was used and this was done with the open-source statistical package R. For this analysis, the estimate, its standard deviation, and the *t* value are provided. A *p* value < 0.05 was considered statistically significant.

## Results

Basic patient characteristics are provided in Table 1. The median gestational age at delivery was 32 completed weeks (IQR 29–33). The median estimated fetal weight was 1520 g (IQR 1049–1758). The median time interval between the last sonographic biometry and delivery was eight days (IQR 4–12). At delivery, there was a median actual birth weight of 1530 g (IQR 1103–1820). In reference to the Hadlock’s formula, a median discrepancy between the sonographically estimated fetal weight and the actual birth weight was 106 g (IQR 56–190). The percentage error and its standard deviation were − 2.5 ± 12.1%, and the median percentage error was − 3.6%.

**Table 1:** Basic patient characteristics

Age (years)^a^	32 (27; 35)
Body mass index (kg/m^2^)^a^	23.0 (21.5; 26.2)
Pregnancy after IVF treatment^b^	67 (69.1)
Parity^b^	
0	72 (74.2)
1	18 (18.6)
≥ 2	7 (7.2)
Cigarette smoking during pregnancy^b^	14 (14.4)
Chorionicity^b^	
1	4 (4.1)
2	31 (32.0)
3	62 (63.9)
Amniocity^b^	
1	0
2	0
3	97 (100)

Data are presented as ^a^median (interquartile range) or ^b^numbers (frequencies)

In reference to the Schild’s formula, whereby 64 children had to be excluded with fetal birth weight < 1000 g, a median discrepancy between the sonographically estimated fetal weight and the actual birth weight was 14.3 g (IQR − 107.3 to 157.5). The percentage error and its standard deviation were + 2.2% ± 13.0, and the median percentage error was 0.8%.

To focus on the predictive power of sonographic fetal weight estimation, and to evaluate other factors that could possibly influence the reliability of estimated fetal weight, we tested parameters predictive of actual birth weight in generalized linear models. Details of this analysis for Hadlock’s formula are provided in Table 2. Estimated fetal weight was the most important factor predictive of actual birth weight, with an estimate of 0.920 (*p *< 0.001), which means that, per estimated gram, there were 0.92 g of actual birth weight, on average, suggesting a slight overall overestimation. As a second numeric parameter, the time interval between sonographic weight estimation and delivery was of significant relevance: per day, 19.522 g of actual birth weight should have been taken into account (*p *< 0.001). Concerning nominal variables and gestational age at delivery ≥ 28 completed weeks were associated with a mean estimate of + 75.653 g per fetus (*p *= 0.045), whereas, female neonates had been slightly but significantly mis-estimated by a mean estimate of − 50.473 g per fetus (*p *= 0.005). Chorionicity, parity, maternal age, and the presence of gestational diabetes mellitus were not found to have been of significant influence. A similar model was calculated using Schild’s formula for fetal weight estimation (Table 3). This analysis led to comparable results: estimated fetal weight (0.82, *p* < 0.001), fetal sex (− 95.85, *p* < 0.001), and the time interval between sonographic weight estimation and delivery (21.94, *p* < 0.001) were significantly predictive for birth weight. Another focus of interest was the accuracy of the prediction of SGA neonates by means of Hadlock’s sonographic weight estimation. As mentioned above, two different reference populations [19, 20] were used for the definition of SGA by an estimated or actual weight < 10th percentile. When using the Canadian Perinatal Surveillance System reference population [19], six/291 fetuses (2.1%, i.e., two pregnancies) had to be excluded, since sonographic estimation had been performed in the completed 21 and 22 weeks of gestation, respectively, and no reference values are available for these gestational ages. Similarly, nine fetuses (3.1%, i.e., three pregnancies) had to be excluded when using the German triplet reference population [20]. For both approaches, the sonographic prediction of SGA was significantly reliable (*p* < 0.001), with positive and negative predictive values ranging from 81.3 to 100.0%. Details of these analyses are provided in Table 4.

**Table 2:** Generalized linear model for the evaluation of factors that influence the discrepancy between estimated fetal weight and actual birth weight (in g), *n* = 291

Coefficient	Estimate	Standard deviation (estimate)	*t* value	*p*
Intercept	33.925	66.621	0.509	0.611
Estimated fetal weight using Hadlock’s formula (g)	0.920	0.027	33.501	< 0.001*
Maternal age (years)	− 2.783	1.610	− 1.729	0.085
Parity (*n*)	− 3.344	14.623	− 0.229	0.819
Chorionicity: 3 (versus 1 and 2)	7.228	9.292	0.778	0.437
Presence of gestational diabetes mellitus	29.361	21.618	1.358	0.176
Gestational age at delivery ≥ 28 completed weeks (versus ≤ 27 completed weeks)	75.653	37.554	2.014	0.045*
Fetal sex: female (versus male)	− 50.473	17.794	− 2.837	0.005*
Time interval between sonographic fetal weight estimation and delivery	19.521	2.192	8.905	< 0.001*

Multiple *R*^2^ = 0.927, adjusted *R*^2^ = 0.924

*Significant results are presented in italic type

**Table 3:** Generalized linear model for the evaluation of factors that influence the discrepancy between estimated fetal weight and actual birth weight (in g), *n* = 226 fetuses

Coefficient	Estimate	Standard deviation (estimate)	*t* value	*p*
Intercept	154.00	107.33	1.434	0.153
Estimated fetal weight using Schild’s formula (g)	0.82	0.04	21.505	< *0.001*
Maternal age (years)	− 2.44	2.11	− 1.159	0.248
Parity (*n*)	− 21.35	18.19	− 1.174	0.242
Chorionicity: 3 (versus 1 and 2)	27.46	20.75	1.324	0.187
Presence of gestational diabetes mellitus	48.16	25.52	1.887	0.060
Fetal sex: female (versus male)	− 95.85	23.79	− 4.030	<* 0.001*
Time interval between sonographic fetal weight estimation and delivery	21.94	2.58	7.696	<* 0.001*

Multiple *R*^2^ = 0.743, adjusted *R*^2^ = 0.735

*Significant results are presented in italic type. Parameter “Gestational age at delivery ≥ 28 completed weeks (versus ≤ 27 completed weeks)” was excluded due to lack of data—all fetuses < 1000 g had to be excluded from this analysis

**Table 4:** Accuracy of small-for-gestational-age fetuses with sonographic diagnosis of fetal growth restriction (Hadlock formula)

Statistical parameter	Value	95% confidence interval	*p*
Reference population: Canadian Perinatal Surveillance System singleton growth curves [17]			
Odds ratio	34.7	7.3–226.4	< 0.001
Sensitivity (%)	24.2	14.5–36.4	
Specificity (%)	99.1	96.7–99.9	
Positive predictive value (%)	88.9	65.3–98.6	
Negative predictive value (%)	81.3	76.1–85.8	
Reference population: German Percentile Values for the Anthropometric Dimensions of Triplet Neonates [18]			
Odds ratio	1733.4	15.3–111,665.8	< 0.001
Sensitivity (%)	26.3	12.7–41.2	
Specificity (%)	100.0	98.1–100.0	
Positive predictive value (%)	100.0	62.8–100.0	
Negative predictive value (%)	89.2	84.7–92.5	

Finally, we analyzed whether an inter-triplet growth discordance > 25% could have been reliably predicted using Hadlock’s sonographic fetal weight estimation. The last biometry before delivery had suggested that 14/97 triplet pregnancies (14.4%) would have resulted in such a growth discordance, whereas it was actually the case in 23/97 cases (23.7%). Prediction had been possible with an odds ratio of 21.7 (95% CI 4.6–117.2), a sensitivity of 47.8% (95% CI 26.8–69.4), a specificity of 95.9% (95% CI 88.6–99.2), a positive predictive value of 78.6% (95% CI 49.2–95.3), and a negative predictive value of 85.5% (95% CI 76.1–92.3; *p *< 0.001).

## Comment

Because triplet pregnancies are often associated with complications, which includes early preterm delivery in many cases [7]; accurate prenatal sonographic assessment is essential. Nonetheless, only few studies have addressed the reliability of fetal weight estimation in these high-risk pregnancies as yet [8, 11–13]. To the best of our knowledge, the data set presented herein is the largest about the specific topic of the accuracy of fetal weight estimation in triplets.

Notably, this retrospective study revealed that sonographic fetal weight estimation is considerably accurate, with a median discrepancy between the estimated fetal weight and the actual birth weight of 106 g. Three other factors added to the prediction of the actual birth weight and these were (Table 2): (i) the time interval between biometry and delivery. It seems reasonable that, with every passing day, the actual birth weight will increase. This was the fact, with a mean of nearly 20 g per day. Although the generalized linear model could adjust for this factor, the estimated fetal weights and the corresponding actual birth weights were not available on the same day. However, the time median interval reported by the most recent study on fetal weight estimation in triplets, published by Sclar et al. in 2017, was similar (8 days) [13]. Moreover, this obviously reflects the circumstances in the clinical routine. (ii) Another factor that contributed to the predictive model was fetal sex. In our data set, female neonates had been overestimated by a mean of 50.473 g per fetus. It has been reported that male fetuses reveal increased fetal size as early as in the first trimester [21]. Moreover, a large retrospective comparison of multiple formulas for sonographic fetal weight estimation revealed that their accuracy was significantly related to fetal sex, and that, in addition, it might be valuable to use different formulas for weight estimation in female and male fetuses [22]. (iii) Last but not least, gestational age at delivery added to fetal weight estimation: according to our model, for pregnancies delivered at ≥ 28 completed weeks of gestation, a mean of 75.6 g would have to be added to the fetal weight estimation to achieve higher accuracy. This is a notable finding, keeping in mind that, in a recent review on factors that influence the accuracy of fetal weight estimation in preterm deliveries, gestational age was not of significant influence [23]. However, it seems obvious that, the more a fetus weighs at a higher gestational age, the higher is the statistical risk to incorrectly estimate the actual birth weight. This shows the limitations of the generalized linear model used. The model provides accurate information about predictive factors that were of clinical relevance. However, it cannot assess the extent to which some of these factors influence fetal weight estimation in a clinically reliable way, which is also attributable to the small sample size, a typical limiting factor in triplet studies. Moreover, Hadlock’s formula was used in the present study, which might not represent the most accurate formula available [22] despite its widespread use. However, all previous studies on the accuracy of fetal weight estimation in triplets have used Hadlock’s formula [8, 11–13].

Notably, the use of Schild’s formula for fetal weight estimation which is sex-specific did not completely eliminate the influence of fetal sex on the discrepancy between estimated fetal weight and actual birth weight (Table 3). Moreover, one could argue that the multivariate model using Schild’s formula was associated with a lower explanatory power (adjusted *R*^2^ = 0.735 versus adjusted *R*^2^ = 0.924 for Hadlock’s formula). However, neither Hadlock’s nor Schild’s formula has been designed for multiple pregnancies and might, therefore, not be an optimal way for fetal weight estimation in these pregnancies. In addition, the analysis including Schild’s formula comprised less cases which might have contributed to the lower explanatory power. Nonetheless, this also shows the need to find better formulas for fetal weight estimation, particularly in special conditions such as multiple pregnancies or growth-restricted fetuses [10].

The next focus of the present study was the accuracy of the prediction of SGA neonates. It seems reasonable that this would also depend on the definition of SGA, and, thus, on the reference population used. The two different reference populations used herein to define the 10th percentile were the Canadian Perinatal Surveillance System singleton growth curves [19] and the German Percentile Values for the Anthropometric Dimensions of Triplet Neonates [20]. The use of these populations could be questioned, but local current reference populations were needed. Whether singleton- or triplet-derived reference populations should be used remains open and more clinical experience needs to be gained [20]. However, in both cases, SGA could be predicted with specificities and positive predictive values of about 90–100% and negative predictive values of about 80–90%, which we consider quite reliable.

The same was true for the prediction of inter-triplet growth discordance > 25%. With a specificity, a positive and a negative predictive value of nearly 96, 79, and 86%, respectively, the results derived in our data set were quite similar to those of Sclar et al. (94.1, 66.7, and 97.1%, respectively) [13]. However, keeping in mind that birth-weight discordance among multiple pregnancies is highly suggestive of an adverse neonatal outcome [24], even higher reliability would be desirable. It is possible that this could be achieved with the use of more accurate formulas for weight estimation and with adequate training, probably with a specialization in multiple pregnancy sonography.

The limitations of this study are its retrospective design and the potential source of error when correlating each prenatally examined fetus with the actual birth order. However, we consider the large sample size and that, all ultrasound examinations had been performed by highly experienced obstetricians who were all members of the clinical working group for multiple pregnancies, which we believe contributed to the high overall accuracy of fetal weight estimation, as a strength. Again, the examiner’s level of experience might be an important factor [23]. Moreover, to the best of our knowledge, the present data set is the largest for the accuracy of weight estimation in triplets thus far.

In conclusion, fetal weight estimation in triplets, using Hadlock’s formula, can be quite accurate with a percentage error of − 2.5 ± 12.1%, at least when performed by experts for multiple pregnancies. However, there is room for further improvement. This might be achieved by applying other formulas than that provided by Hadlock et al. [17], probably by including important factors that influence fetal weight estimation, and first and foremost among these factors is fetal sex [23]. These clinical issues, especially the search for an optimized formula for fetal weight estimation including all surrounding factors, should be the focus of future studies on fetal weight estimation in triplets.
